# Lymphocytes subset composition and modulation of sphingosie-1-phosphate mediated migration in multiple sclerosis patients treated with fingolimod or interferon-β

**DOI:** 10.1186/1479-5876-10-S3-P18

**Published:** 2012-11-28

**Authors:** Heiko Sic, Michael Stock, Sebastian Rauer, Hermann Eibel

**Affiliations:** 1 Centre of Chronic Immunodeficiency, University Medical Center, Freiburg, Germany; 2 Dept. of Neurology, University Medical Center, Freiburg, Germany

## 

Lymphocytes strongly contribute to the pathology of multiple sclerosis (MS). Thereby, frequently activated or reactivated autoreactive lymphocytes differentiate into effector cells that enter the central nervous system (CNS) causing tissue inflammation and damage. In order to reach the CNS, migratory signals allowing the entry into the circulatory system are vital. Sphingosine-1-phosphate (S1P) is a key mediator of lymphocyte egress that binds to five S1P receptors (S1P1-5). The immunomodulatory drug fingolimod (FTY720, Gilenya) directly targets S1P receptors and inhibits lymphocyte egress, whereas the exact effector function of interferon-β (IFN-β) in MS therapy remains elusive. However, IFN-β is known to induce CD69 expression, which by direct interaction, downmodulates S1P1 surface expression preventing lymphocyte egress. Since the block of lymphocyte egress is not complete, we analyzed subset composition and migratory responsiveness towards S1P of the lymphocytes remaining in the circulation of MS patients treated with fingolimod or IFN-β. Here we show that fingolimod treatment increased the responsiveness of the remaining T-lymphocytes towards S1P and that B cell migration of IFN-β treated patients changed from weak to normal. We found changes in the subset composition and absolute numbers in both treatment groups. While fingolimod reduced the number of almost all analyzed subsets in the circulation IFN-β specifically reduced the numbers of marginal zone and memory B cells.

In conclusion, we think that by reducing “memory” type lymphocytes in the circulation fingolimod and IFN-β impair reactivation of autoreactive cells. In addition, treatment might inhibit the egress of newly formed effector cells and selects for stronger S1P responsive cells. Taken together these effects contribute to an overall beneficial outcome.

